# Effects of digital Cognitive Behavioural Therapy for Insomnia on cognitive function: study protocol for a randomised controlled trial

**DOI:** 10.1186/s13063-017-2012-6

**Published:** 2017-06-17

**Authors:** Simon D. Kyle, Madeleine E. D. Hurry, Richard Emsley, Annemarie I. Luik, Ximena Omlin, Kai Spiegelhalder, Colin A. Espie, Claire E. Sexton

**Affiliations:** 1 Nuffield Department of Clinical Neurosciences, Sleep and Circadian Neuroscience Institute, University of Oxford, Sir William Dunn School of Pathology, South Parks Road, Oxford, OX1 3RE UK; 2 Oxford Nuffield Department of Clinical Neurosciences, Centre for Functional Magnetic Resonance Imaging of the Brain (FMRIB Centre), University of Oxford, Oxford, UK; 3 Centre for Biostatistics, School of Health Sciences, The University of Manchester, Manchester Academic Health Science Centre, Manchester, UK; 4 Big Health Ltd., London, UK; 5 Department of Psychiatry and Psychotherapy, Medical Center - University of Freiburg, Faculty of Medicine, University of Freiburg, Freiburg, Germany

**Keywords:** Sleep, Insomnia, Cognitive impairment, Cognitive behavioural therapy, Internet

## Abstract

**Background:**

The daytime effects of insomnia pose a significant burden to patients and drive treatment seeking. In addition to subjective deficits, meta-analytic data show that patients experience reliable objective impairments across several cognitive domains. While Cognitive Behavioural Therapy for Insomnia (CBT-I) is an effective and scalable treatment, we know little about its impact upon cognitive function. Trials of CBT-I have typically used proxy measures for cognitive functioning, such as fatigue or work performance scales, and no study has assessed self-reported impairment in cognitive function as a primary outcome. Moreover, only a small number of studies have assessed objective cognitive performance, pre-to-post CBT-I, with mixed results. This study specifically aims to (1) investigate the impact of CBT-I on cognitive functioning, assessed through both self-reported impairment and objective performance measures, and (2) examine whether change in sleep mediates this impact.

**Methods/design:**

We propose a randomised controlled trial of 404 community participants meeting criteria for Insomnia Disorder. In the DISCO trial (***D*** *efining the* ***I*** *mpact of improved* ***S*** *leep on* ***CO*** *gnitive function (DISCO)*) participants will be randomised to digital automated CBT-I delivered by a web and/or mobile platform (in addition to treatment as usual (TAU)) or to a wait-list control (in addition to TAU). Online assessments will take place at 0 (baseline), 10 (post-treatment), and 24 (follow-up) weeks. At week 25, all participants allocated to the wait-list group will be offered digital CBT-I, at which point the controlled element of the trial will be complete. The primary outcome is self-reported cognitive impairment at post-treatment (10 weeks). Secondary outcomes include objective cognitive performance, insomnia severity, sleepiness, fatigue, and self-reported cognitive failures and emotional distress. All main analyses will be carried out on completion of follow-up assessments and will be based on the intention-to-treat principle. Further analyses will determine to what extent observed changes in self-reported cognitive impairment and objective cognitive performance are mediated by changes in sleep. The trial is supported by the National Institute for Health Research (NIHR) Oxford Biomedical Research Centre (BRC) based at Oxford University Hospitals NHS Trust and University of Oxford, and by the NIHR Oxford Health BRC.

**Discussion:**

This study will be the first large-scale examination of the impact of digital CBT-I on self-reported cognitive impairment and objective cognitive performance.

**Trial registration:**

ISRCTN, ID: ISRCTN89237370. Registered on 17 October 2016.

**Electronic supplementary material:**

The online version of this article (doi:10.1186/s13063-017-2012-6) contains supplementary material, which is available to authorized users.

## Background

Insomnia disorder is defined as persistent difficulties with sleep initiation and/or maintenance, resulting in significant daytime impairment. Insomnia affects approximately 10–12% of the adult population and is associated with increased risk for cardiovascular disease, depression, and early mortality [1, 2]. Daytime functioning and quality of life are known to be severely affected in those with insomnia and often drive treatment seeking [3–5]. More specifically, previous work shows that the most commonly cited areas of daytime dysfunction are problems with fatigue, work performance, cognitive functioning, and emotional regulation [6].

With respect to cognitive function, meta-analytic data indicate that patients exhibit reliable impairments (of medium effect size) in tasks probing episodic memory, working memory, and problem solving [7]. Recent, well-controlled studies also find evidence of insomnia-related impairments in switching of attention and working memory [8], and sustained attention and episodic memory [9]. These impairments are associated with global insomnia severity, sleep duration, and disturbed sleep continuity (from both sleep diaries and polysomnography). In general, neurocognitive findings are consistent with results from functional imaging studies, which reveal evidence of hypoactivation within fronto-striatal networks during task-related functional magnetic resonance imaging (fMRI) [10–13].

Despite cognitive impairment featuring in contemporary diagnostic criteria for insomnia (Diagnostic and Statistical Manual of Mental Disorders, 5th edition: DSM-5; International Classification of Sleep Disorders, 3rd edition: ICSD-3) [14], and posing significant burden to patients, there has been little evaluation of the impact of insomnia treatments on cognitive function [15]. This is surprising because cognitive impairment may drive other daytime impairments associated with insomnia (e.g. reduced work performance and increased accident risk) [6, 16]. To date, trials of Cognitive Behavioural Therapy for Insomnia (CBT-I), the first-line treatment for persistent insomnia disorder [1], have tended to employ proxy measures (e.g. general daytime functioning, work performance) or nonvalidated, single items to probe self-reported cognitive functioning as a secondary outcome [15]. No study has assessed cognitive functioning as a primary outcome. Moreover, to our knowledge only four small studies have assessed *objective cognitive performance*, pre-to-post CBT-I [10, 17–19], providing preliminary evidence that sleep improvement may be associated with improvements in complex attention and executive function. However, the largest trial to date [19] failed to observe treatment effects for delayed recall, abstract reasoning, or working memory. Thus, it remains unclear if CBT-I confers reliable improvement to cognitive performance and to what extent such improvement may be mediated by reductions in insomnia severity. If insomnia causally drives cognitive impairment then we might logically expect that (1) CBT-I improves both sleep and cognitive function, and (2) treatment effects on cognition will operate via the pathway of improved sleep.

The aim of this study, therefore, is to investigate the impact of CBT-I on cognitive functioning, assessed through both self-report and objective measures. We will recruit participants meeting criteria for insomnia disorder who also endorse cognitive complaints, and conduct a parallel-group, randomised controlled trial (RCT) of CBT-I + TAU versus wait-list control (WLC + TAU). CBT-I will be delivered online via a website and/or app [20]. Online interventions have shown robust effects on insomnia severity and self-reported sleep parameters, with effect sizes in the range of face-to-face interventions [21, 22]. Assessments, including questionnaires and computerised cognitive tasks, will also be delivered and completed fully online. This will be the first examination of the impact of digital CBT-I (dCBT-I) on self-reported cognitive impairment and objective performance in participants meeting criteria for insomnia disorder.

The primary hypothesis for the trial is:

1. dCBT-I will reduce self-reported cognitive impairment at the end of treatment (10 weeks) relative to WLC

The secondary hypotheses are:

1. dCBT-I will reduce self-reported cognitive impairment at follow-up (24 weeks) relative to WLC
2. dCBT-I will improve objective cognitive performance (in the following domains: simple attention, visual attention, episodic memory, working memory, and complex processing speed), relative to WLC (10 and 24 weeks)
3. dCBT-I will reduce insomnia severity and improve sleep efficiency relative to WLC (10 and 24 weeks)
4. Change in insomnia severity and sleep efficiency at week 10 will mediate change in self-reported cognitive impairment and objective cognitive performance at week 24
5. dCBT-I will lead to improvements in fatigue, sleepiness, self-reported cognitive failures, depression, and anxiety relative to WLC (weeks 10 and 24)

## Methods/design

### Research design

**Fig. 1:**
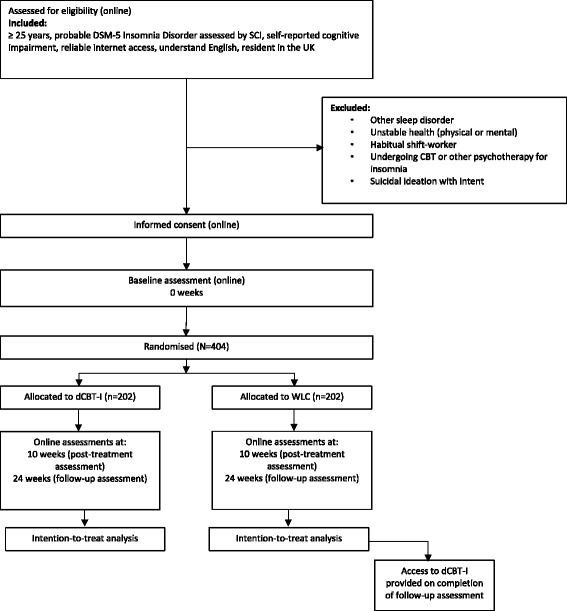
Flow chart diagram showing summary of the trial design for the Defining the Impact of improved Sleep on COgnitive function (DISCO) study. *DSM-5 Diagnostic and Statistical Manual of Mental Disorders, version 5*, *SCI* Sleep Condition Indicator, *dCBT-I* digital Cognitive Behavioural Therapy for Insomnia, *WLC* Wait-list control

The study is a parallel-group, superiority RCT of dCBT-I (+ TAU) versus WLC (+ TAU). The study will be carried out completely online. Participants will be administered screening, informed consent, assessments (tasks and questionnaires), allocation to condition, and intervention through web-based platforms. Online assessment of our primary and secondary dependent variables will take place at 0 (baseline), 10 (post-treatment), and 24 (follow-up) weeks. At week 25 all participants allocated to the WLC will be offered dCBT-I to improve their insomnia (see Fig. 1 for trial design). Ethical approval has been granted from the University of Oxford Medical Sciences Inter-Divisional Research Ethics Committee (R46116/RE001) and the trial is registered at ISRCTN (ISRCTN89237370).

### Participants

We will recruit 404 community participants who report clinically-significant insomnia. Our inclusion criteria comprise: (a) a positive screen for probable DSM-5 insomnia disorder using items from the Sleep Condition Indicator (SCI) (scoring ≤2 on item 1 (sleep latency) *or* item 2 (wakefulness during the night) + scoring ≤2 on item 3 (frequency of disturbance) + scoring ≤1 on item 4 (sleep quality) + scoring ≤2 on daytime functioning items 5 *or* 6 + scoring ≤2 on item 8 (chronicity of problem)) [23], (b) endorsement of difficulties with concentration or memory using items from the Daytime Functioning and Sleep Attribution Scale (selecting ‘somewhat of a problem’ or ‘a very big problem’ on at least one item, when asked how much of a problem ‘difficulty concentrating and focussing on things’ and ‘difficulty remembering things’ has been in the past 2 weeks) [24], (c) being aged 25 years and above (to minimise the possibility that circadian rhythm disruption, which is common in late adolescence/early 20s, is the source of insomnia complaints), (d) having reliable Internet access at home or at work, (e) being able to read and understand English, and (f) currently living in the UK. We will screen for comorbid conditions and medication use via online survey and exclude people with (1) symptoms of a probable additional sleep disorder (e.g. possible obstructive sleep apnoea, restless legs syndrome [25]), (2) diagnosis of mild cognitive impairment or dementia, (3) psychosis or mania, (4) serious physical health concerns necessitating surgery or with a prognosis of under 6 months, (4) those undergoing a psychological treatment programme for insomnia with a health professional, (5) habitual night shift, evening, or rotating shift-workers, (6) those taking prescribed sleeping pills more than 2 nights in the past 2 weeks prior to study entry, and (7) those with suicidal ideation with intent. We will not omit participants for any other physical or mental health problems. The study will recruit through multiple channels. These include online, print and broadcast media advertisements, and the use of contact lists where adults who have agreed to be contacted about future studies will be notified about the DISCO trial. In addition to receiving free access to the dCBT-I programme, participants will also receive payment in the form of Amazon™ gift vouchers for completing each assessment point (£5 for baseline; £10 for post-treatment; £15 for follow-up).

### Randomisation and allocation concealment

This study will use simple randomisation with an allocation ratio of 1:1, as recommended for large clinical trials [26]. Randomisation will be carried out using the randomisation function within Qualtrics Survey Software (Qualtrics, Provo, UT, USA). On completion of baseline measures, each subject will be randomly assigned by the software to either dCBT-I or WLC. The research team, therefore, will be unable to influence randomisation and will have no access to future allocations.

### Blinding

Self-report assessments and performance tasks will be completed entirely online. Participants will be informed of their randomisation outcome (dCBT-I or WLC) via email, and so they will not be blind to treatment allocation. The research team, except the trial coordinator, will be blind to allocation. The trial coordinator will not be blind since they will inform participants of group allocation and monitor completion of assessments. Participant contact with the trial coordinator will be limited to scripted phrases, detailing instructions for dCBT-I access and completion of assessments, and will not cover therapy content or support. Statistical analyses will be conducted by a member of the research team who will have access to all data.

### Assessment points

**Fig. 2:**
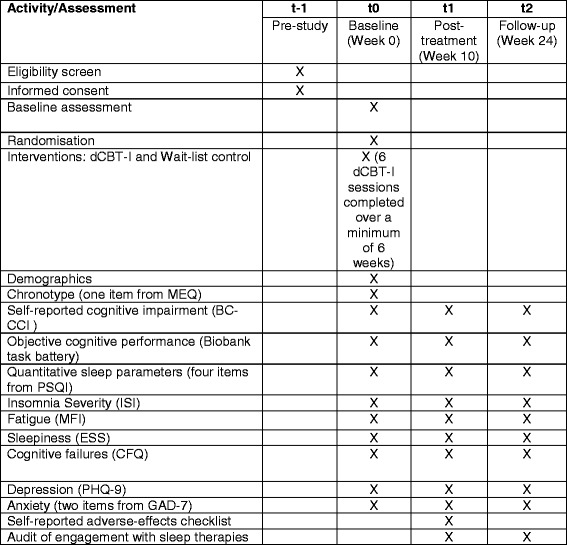
Standard Protocol Items: Recommendations for Interventional Trials (SPIRIT) figure

Assessments will take place at weeks 0 (baseline), 10 (post-treatment), and 24 (follow-up) (see Fig. 2). Participants are advised to allow enough time to complete the questionnaires and cognitive tasks in one session, on their home computer or laptop in a quiet, distraction-free environment. On completion of the trial (week 25), all participants in the control arm will be offered access to dCBT-I. Baseline variables include demographic information such as age, gender, ethnic group, partner status, employment status, and education. Frequency of sleep medication use will also be monitored at each assessment point.

### Intervention

dCBT-I will be delivered using the Sleepio® programme (www.sleepio.com and associated Sleepio® app) [19]. We have previously described the Sleepio® programme in two trial protocols published in this journal [27, 28]. The programme is fully automated and its underlying algorithms drive the delivery of information, support, and advice in a personally tailored manner. Delivery is structured into six weekly sessions, lasting an approximately 20 min each. The full programme can be accessed via the website or iOS app. Treatment content is based on CBT-I manuals and includes a behavioural component (sleep restriction, stimulus control, and relaxation), a cognitive component (paradoxical intention, cognitive restructuring, mindfulness, positive imagery, and putting the day to rest) and an educational component (psychoeducation and sleep hygiene) [29–31].

The programme is highly interactive, and content is presented by an animated virtual therapist (‘The Prof’). Before the start of the programme, participants complete a questionnaire to tailor the therapy and to set treatment goals. Participants complete daily sleep diary information throughout the intervention, which is used by the programme to provide tailored, personalised advice. Participants can opt to receive an email and/or SMS reminder each morning to prompt them to fill in their sleep diary. In addition, throughout the course of therapy, participants have access to an online community forum; weekly sleep expert sessions moderated by a clinical psychologist; and online library of information about sleep and sleep disorders. During weekly sleep expert sessions, users may vote on topics and submit questions to be addressed by the clinical psychologist. Questions are answered in such a way as to benefit as many people as possible and no personal medical advice is provided. Participants can view their online ‘case file’, which includes four sections: a progress review, a 'to-do' list, an agreed sleep schedule, and a list of further reading. The system provides online analytics which can be used to monitor adherence by assessing how many sessions were completed and the number of weeks to complete the course. All information gathered during the programme will be stored in encrypted form on secure servers. Participants will have access to the intervention for up to 12 weeks. dCBT-I will in effect be dCBT-I + TAU because there will be no requirement for participants (in either group) to alter their usual care in any way.

### Outcome measures

Our primary outcome will be *self-reported cognitive impairment*, assessed using the British Columbia Cognitive Complaints Inventory (BC-CCI) [32]. The BC-CCI comprises six questions scored using a 4-point scale (0–3 points; total score range: 0–18) probing perceived cognitive problems (with concentration, memory, and thinking skills) during the past 7 days. It was originally designed to probe cognitive complaints in those with depression – a disorder highly comorbid with insomnia, and sharing similar cognitive features [33]. Given the absence of a widely used measure of insomnia-related cognitive impairment the BC-CCI was selected based on good face validity, internal consistency data (Cronbach’s alpha = 0.82–0.86), and short (1-week) reference period.

### Secondary measures

*Objective cognitive performance* will be assessed through an online battery of tasks and will include measures of simple attention (*dependent variable [DV]: simple reaction time [msec] for correct identification of matching pairs [‘snap’]*), episodic memory (*DV: number of attempts to identify identical pairs of cards within a matrix)*, working memory (*DV: longest sequence of correctly recalled digits*), visual attention (*DV: time taken [secs] to link numbered circles*), and complex processing speed (*DV: number of correctly matched digit-symbols within a 2-min interval*). The battery was developed by the UK Biobank Cognitive Psychology Sub-Group for Cognitive Assessments. Prior to task commencement, participants are asked which text entry and pointing methods they will use to complete tasks (e.g. on full-sized keyboard, touchscreen, laptop trackpad or mouse).

#### Insomnia severity

Participants will complete the Insomnia Severity Index (ISI) [34] to quantify global insomnia severity. The ISI is a seven-item insomnia assessment tool, probing both nighttime and daytime aspects of insomnia disorder, and is sensitive to change following CBT-I. It is a recommended outcome measure in insomnia trials [35]. The ISI will be supplemented with four items from the Pittsburgh Sleep Quality Index (PSQI) [36] to permit calculation of quantitative sleep parameters, namely: total time in bed, sleep-onset latency, total sleep time, and *sleep efficiency*.

#### Fatigue

Fatigue will be assessed with the Multidimensional Fatigue Inventory (MFI) [37], a 20-item measure comprising five subscales; general fatigue, physical fatigue, mental fatigue, reduced motivation and reduced activity.

#### Sleepiness

Daytime somnolence will be assessed with the Epworth Sleepiness Scale (ESS) [38], an eight-item measure (score range: 0–24) probing propensity for ‘dosing’ in a range of daytime situations.

#### Cognitive failures

Self-reported cognitive failures will be assessed with the Cognitive Failures Questionnaire (CFQ) [39]. The CFQ comprises 25 questions scored using a 5-point Likert scale (range: 0–100) to identify frequency of minor cognitive mistakes over the past month.

#### Depression

Depressive symptoms will be measured using the nine-item (score range: 0–27) Patient Health Questionnaire (PHQ-9) [40].

#### Anxiety

Symptoms of anxiety will be assessed using two items [‘*Feeling nervous, anxious, or on edge’*, ‘*not being able to stop or control worrying’*; score range: 0–6] from the seven-item Generalised Anxiety Disorder 7 questionnaire (GAD-7) [41].

### Assessment of safety

The likelihood of serious adverse events occurring during this trial is low since CBT-I (in any format) has not been reported to cause them. The intervention offered in the trial has previously been tested in a randomised, placebo-controlled trial and no adverse outcomes were reported [20]. Since the trial is completed fully online, without formal participant contact, we are unlikely to become aware of potential adverse events. However, should we do so, we will define serious adverse events as: (1) death, (2) suicide attempt, (3) admission to hospital, and (4) formal complaints about the online intervention.

Studies have shown that daytime sleepiness and vigilance impairment may increase during Sleep Restriction Therapy (SRT) (one component of CBT-I), owing to restricted sleep opportunity. At the end of treatment (week 10), we will, therefore, ask participants to complete an adapted version of a previously used measure to assess differential rates of self-reported adverse effects [42].

### Sample size calculation

Our planned primary intention-to-treat analysis will compare dCBT-I + TAU versus WLC + TAU for self-reported cognitive impairment (BC-CCI). A systematic review of the cognitive functioning and CBT-I literature shows a standardised mean difference of 0.42 at post-treatment for self-reported outcomes [15]. We will recruit and randomise 404 participants (202 participants/arm). This sample size will give 90% power to detect a minimum standardised effect size of 0.42 at 5% level of significance, accounting for 40% attrition.

#### Statistical analysis

In accordance with Consolidated Standards of Reporting Trials (CONSORT) guidelines, we will record and report all participant flow [43]. Descriptive statistics of recruitment, dropout, and completeness of interventions will be provided. The main efficacy analysis will be via intention-to-treat including all participants, with no planned interim analysis for efficacy or futility. Baseline characteristics will be presented by randomised group without formal statistical tests. We will test the primary hypothesis for between-group change in the primary outcome (BC-CCI) at 10 weeks using analysis of covariance with baseline outcome measure and treatment assignment as fixed effects, and apply standard regression diagnostics. The analysis will use statistical techniques for handling missing outcome data under a missing-at-random assumption. Secondary outcomes will be analysed using an analogous method. Analysis of all treatment effects will be undertaken after follow-up (week 24) assessments are completed. Additional exploratory analyses will assess whether age moderates treatment-related effects on cognitive functioning outcomes.

We will use modern causal inference methods to investigate the mediation hypothesis. If the efficacy analysis shows significant between-group differences in the ISI and sleep efficiency (based on PSQI items) at 10 weeks, then we will use parametric regression models to test for the indirect effect of ISI/sleep efficiency on primary and secondary cognitive outcomes at 24 weeks, and the residual direct effect of treatment on cognitive outcomes at 24 weeks. Since all the measures are continuous, the indirect effects are calculated by multiplying relevant pathways and bootstrapping is used to produce valid standard errors for the indirect effects. All analyses will adjust for baseline measures of the mediators (ISI/sleep efficiency), outcomes and putative measured confounders. Mediation analyses are potentially biased by measurement error in mediators and hidden confounding between mediators and outcomes and we will investigate the sensitivity of the estimates to these problems. All analyses will be carried out using Stata [44].

### Dissemination

We will publish the results of this study in peer-reviewed journals, irrespective of magnitude or direction of effect. Findings will also be presented at both national and international scientific meetings. The results will be made available online wherever possible, if permitted by journal policies.

Items in this protocol comply with the Standard Protocol Items: Recommendations for Interventional Trials (SPIRIT) Checklist (see SPIRIT Checklist in Additional file 1 and Fig. 2 for the SPIRIT figure).

## Discussion

Insomnia is a prevalent, persistent and impairing sleep disorder. Patients consider cognitive dysfunction as one of the most important consequences of insomnia, yet we do not know whether such dysfunction can be ameliorated through evidence-based intervention. This study will determine whether treating insomnia with dCBT-I improves both self-reported cognitive impairment and objective cognitive performance, and whether change in insomnia severity and sleep efficiency mediates such improvement.

### Trial status

Recruitment commenced on 18 October 2016 and is ongoing.

## Additional file

Additional file 1: SPIRIT Checklist. (DOCX 51 kb)

## Abbreviations

BC-CCI: British Columbia Cognitive Complaints Inventory
CBT: Cognitive Behavioural Therapy
CBT-I: Cognitive Behavioural Therapy for Insomnia
CFQ: Cognitive Failures Questionnaire
dCBT-I: Digital Cognitive Behavioural Therapy for Insomnia
DSM-5: Diagnostic and Statistical Manual of Mental Disorders,5th edition
DV: Dependent variable
ESS: Epworth Sleepiness Scale
GAD: Generalised Anxiety Disorder
ICSD-3: International Classification of Sleep Disorders,3rd edition
ISI: Insomnia Severity Index
MEQ: Morningness-Eveningness Questionnaire
MFI: Multidimensional Fatigue Index
PHQ-9: Patient Health Questionnaire
PSQI: Pittsburgh Sleep Quality Index
RCT: Randomised controlled trial
SCI: Sleep Condition Indicator
SRT: Sleep restriction therapy
TAU: Treatment as usual
WLC: Wait-list control
